# Supplementary material for: Swimming Behavior of Daphnia magna Is Altered by Pesticides of Concern, as Components of Agricultural Surface Water and in Acute Exposures
Source: Biology (Basel). 2023 Mar 10;12(3):425. doi: 10.3390/biology12030425 (PMC10045752; doi:10.3390/biology12030425)
Supplement: Supplementary file 1 [file biology-12-00425-s001.zip › biology-2168840-supplementary.pdf]

Supplemental data

Table S1. September 2019 (before first flush): Pesticides detected in ambient field water collected from CDPR long-term monitoring sites in Salinas, CA on 9/17/2019. Trace values are below the MDL and ND represents analytes that were not detected.

Analyte	Quail Creek µg/L	Alisal Creek µg/L	Salinas River µg/L
Acetamiprid	0.314	0.137	Trace
Atrazine	ND	ND	ND
Azoxystrobin	0.056	0.029	Trace
Bensulide	3.9	0.888	0.121
Chlorantraniliprole	0.35	0.504	0.021
Chlorpyrifos	ND	0.02	ND
Clothianidin	0.081	0.177	ND
Cyprodinil	ND	Trace	ND
Dimethoate	ND	0.052	ND
Diuron	ND	Trace	ND
Fenamidone	0.247	0.272	ND
Fenhexamid	ND	ND	ND
Fludioxonil	0.148	Trace	ND
Imidacloprid	0.293	0.513	0.014
Indoxacarb	0.146	Trace	ND
Malathion	0.024	0.349	ND
Methomyl	29.9	1.64	0.386
Methoxyfenozide	Trace	0.065	ND
Prometryn	Trace	Trace	ND
Pyraclostrobin	0.112	0.052	ND
Quinoxyfen	ND	ND	ND
Simazine	ND	ND	ND
S-Metolachlor	ND	ND	ND
Thiamethoxam	3.99	0.827	0.064
Trifloxystrobin	ND	ND	ND
Bifenthrin	0.00254	0.00278	ND
Fenpropathrin	ND	0.00530	ND
Lambda Cyhalothrin	0.0797	0.0127	ND
Permethrin Cis	0.108	0.0296	ND
Permethrin Trans	0.126	0.0290	ND
Cyfluthrin	ND	0.00588	ND
Cypermethrin	ND	ND	ND
Esfenvalerate/Fenvalerate	0.0224	ND	ND
Permethrin Total	0.234	0.0586	ND

Table S2. November 2019 (after first flush): Pesticides detected in ambient field water collected from CDPR long-term monitoring sites in Salinas, CA on 11/26/2019. Trace values are below the MDL and ND represents analytes that were not detected.

Analyte	Quail Creek µg/L	Sal_Alisal Creek µg/L	Salinas River µg/L
Acetamiprid	0.19535	0.049	Trace
Atrazine	ND	ND	ND
Azoxystrobin	0.83946	0.08462	Trace
Bensulide	2.5	1.28	Trace
Chlorantraniliprole	0.36458	0.51672	0.04127
Chlorpyrifos	ND	0.12826	ND
Clothianidin	0.89898	0.09285	Trace
Cyprodinil	0.16363	0.02811	Trace
Dimethoate	ND	ND	ND
Diuron	Trace	Trace	0.22422
Fenamidone	0.07757	0.15752	Trace
Fenhexamid	ND	ND	ND
Fludioxonil	0.02429	0.02736	Trace
Imidacloprid	0.30697	0.29254	0.03068
Indoxacarb	0.05475	0.0371	ND
Malathion	0.99858	0.07848	0.02048
Methomyl	0.24669	0.75221	0.07805
Methoxyfenozide	0.05385	0.06433	Trace
Prometryn	Trace	Trace	Trace
Pyraclostrobin	0.18261	0.13099	Trace
Quinoxyfen	Trace	Trace	ND
Simazine	ND	ND	ND
S-Metolachlor	ND	ND	ND
Thiamethoxam	0.28782	0.33599	0.02099
Trifloxystrobin	0.03373	Trace	ND
Bifenthrin	0.0665	0.0308	0.0197
Fenpropathrin	0.0989	0.0297	ND
Lambda Cyhalothrin	0.0324	0.0761	0.00496
Permethrin Cis	0.0182	0.0315	0.00608
Permethrin Trans	0.0146	0.0178	0.00789
Cyfluthrin	ND	0.0162	0.00554
Cypermethrin	ND	ND	ND
Esfenvalerate/Fenvalerate	ND	0.0219	ND
Permethrin Total	0.0328	0.0493	0.0140

Table S3. Change in concentration (µg/L) of analytes of concern, from 9/17/2019 versus 11/26/2019. Analytes which increased in concentration from September to

November are shown in light grey and values that increased and exceeding EPA acute invertebrate aquatic life benchmarks are shown in dark grey. For analytes that were initially detected at Trace levels in September then increased/decreased in November, change value is listed as > 0 or < 0, respectively.

Analyte	△ Quail Creek	△ Alisal Creek	△ Salinas River.
Acetamiprid	-0.12	-0.09	0
Atrazine	0	0	0
Azoxystrobin	0.78	0.06	0
Bensulide	-1.4	0.39	<0
Chlorantraniliprole	0.01	0.01	0.02
Chlorpyrifos	0	0.11	0
Clothianidin	0.82	-0.08	>0
Cyprodinil	0.164	0.028	>0
Dimethoate	0	-0.052	0
Diuron	>0	0	0.224
Fenamidone	-0.17	-0.11	>0
Fenhexamid	0	0	0
Fludioxonil	-0.12	0.027	>0
Imidacloprid	0.01	-0.22	0.02
Indoxacarb	-0.09	0.027	0
Malathion	0.97	-0.27	0.02
Methomyl	-29.65	-0.89	-0.31
Methoxyfenozide	0.054	0	>0
Prometryn	0	0	>0
Pyraclostrobin	0.07	0.08	>0
Quinoxyfen	>0	>0	0
Simazine	0	0	0
S-Metolachlor	0	0	0
Thiamethoxam	-3.7	-0.49	-0.04
Trifloxystrobin	0.034	>0	0
Bifenthrin	0.064	0.028	0.0197
Fenpropathrin	0.0989	0.0297	0
Lambda Cyhalothrin	-0.0473	0.0634	0.00496
Permethrin Cis	-0.0898	0.0019	0.00608
Permethrin Trans	-0.1114	-0.0112	0.00789
Cyfluthrin	0	0.0103	0.00554
Cypermethrin	0	0	0
Esfenvalerate/Fenvalerate	-0.0224	0.029	0
Permethrin Total	-0.2012	-0.0093	0.014

Table S4. Chlorantraniliprole and Imidacloprid chemical concentration confirmations of experimental solutions created for single/binary exposures. Nominal concentrations were 5.0 μg/L for high treatments and 1.0 μg/L for low treatments.

Site	Chlorantraniliprole Result (µg/L)	Imidacloprid Result (µg/L)
CHL LOW	0.986	ND
CHL HIGH	5.71	ND
IMI LOW	ND	0.85
IMI HIGH	ND	4.8
CHL LOW IMI LOW	1.05	0.85
CHL HIGH IMI LOW	5.26	0.947
CHL LOW IMI HIGH	0.996	5.07
CHL HIGH IMI HIGH	5.81	4.93
SOLVENT CONTROL	ND	ND
CONTROL	ND	ND

Table S5. September 2019 (before first flush): Water quality parameters measured using a YSI EXO1 multi-parameter water quality sonde. Parameters recorded include ambient water pH, specific conductance, dissolved oxygen, temperature, alkalinity, hardness, and salinity. Boxes with n/a refer to treatments which were no longer measured due to 100% mortality.

September 2019 Test Initiation

Cond (ms/cm)	DO (mg/L)	Temp (°C)	Alkalinity (CaCO3) mg/L	Hardness (CaCO3) mg/L	Salinity (ppt)
0.967	7.37	20.8	184	376	0.5
0.954	7.05	20.8	108	202	0.5
0.348	8.47	20.2	130	198	0.2

September 2019 48h Water Change

SiteID	pH (units)	Cond (ms/cm)	DO (mg/L)	Temp (°C)	Alkalinity (CaCO3) mg/L	Hardness (CaCO3) mg/L	Salinity (ppt)
Quail Creek	8.17	0.372	7.46	21.8	80	152	0.2
Alisal Creek	8.52	1.356	8.18	21.4	54	192	0.7
Salinas River	8.32	0.525	8.21	21.2	130	136	0.3

September 2019 Test Termination

SiteID	pH (units)	Cond (ms/cm)	DO (mg/L)	Temp (°C)	Alkalinity (CaCO3) mg/L	Hardness (CaCO3) mg/L	Salinity (ppt)

Quail Creek	8.29	0.841	6.63	20.9	190	362	0.4
Alisal Creek	7.91	0.915	6.04	20.7	130	280	0.5
Salinas River	8.32	0.355	6.65	20.3	120	188	0.2

Table S6. September 2019 (before first flush): Results of Repeated Measures ANOVA and Tukey's Multiple comparisons tests for Total Distance Moved, and Photomotor Response. These data sets were then log transformed and analyzed in GraphPad Prism (v8.0) using a one-way ANOVA with a Tukey's Post Hoc test of multiple comparisons.

**September 2019 (before first flush)**			
Repeated Measures ANOVA			
Fixed effects (type III)	P value	F (DFn, DFd)	
Time Bin	0.4061	F (4, 419) = 1.002	
Treatment	**0.0025**	F (3, 419) = 4.839	
Time Bin x Treatment	0.1184	F (12, 419) = 1.507	
Tukey's Multiple Comparison's (Total Distance Moved)			
**Tukey's multiple comparisons test**	**Mean Diff.**	**95.00% CI of diff.**	**Adjusted P Value**
Control 5-10 vs. Hartnell Rd. [12] 5-10	0.1724	-0.1593 to 0.5041	0.926
Control 5-10 vs. Hartnell Rd. [20] 5-10	0.4631	0.1314 to 0.7948	**0.0004**
Control 5-10 vs. Hartnell Rd. [35] 5-10	0.09997	-0.2317 to 0.4317	0.9999
Control 11-15 vs. Hartnell Rd. [12] 11-15	-0.1495	-0.4812 to 0.1823	0.9803
Control 11-15 vs. Hartnell Rd. [20] 11-15	-0.1536	-0.4854 to 0.1781	0.974
Control 11-15 vs. Hartnell Rd. [35] 11-15	-0.2569	-0.5886 to 0.07484	0.3489
Control 16-20 vs. Hartnell Rd. [12] 16-20	-0.06552	-0.3972 to 0.2662	>0.9999
Control 16-20 vs. Hartnell Rd. [20] 16-20	0.4791	0.1474 to 0.8108	**0.0002**
Control 16-20 vs. Hartnell Rd. [35] 16-20	-0.002762	-0.3345 to 0.3289	>0.9999
Control 21-25 vs. Hartnell Rd. [12] 21-25	-0.1076	-0.4393 to 0.2241	0.9996
Control 21-25 vs. Hartnell Rd. [20] 21-25	0.4664	0.1347 to 0.7981	**0.0003**
Control 21-25 vs. Hartnell Rd. [35] 21-25	0.1761	-0.1556 to 0.5078	0.9118
Control 26-30 vs. Hartnell Rd. [12] 26-30	-0.0249	-0.3566 to 0.3068	>0.9999
Control 26-30 vs. Hartnell Rd. [20] 26-30	0.278	-0.05376 to 0.6097	0.2208
Control 26-30 vs. Hartnell Rd. [35] 26-30	0.404	0.07229 to 0.7357	**0.0039**
Control 5-10 vs. Davis Rd. [12] 6-10	0.03617	-0.1226 to 0.1949	>0.9999
Control 5-10 vs. Davis Rd. [20] 6-10	0.04484	-0.1139 to 0.2036	>0.9999
Control 5-10 vs. Davis Rd. [35] 6-10	0.1054	-0.05339 to 0.2642	0.6332
Control 11-15 vs. Davis Rd. [12] 11-15	-0.1791	-0.3379 to -0.02033	**0.012**

Control 11-15 vs. Davis Rd. [20] 11-15	-0.3697	-0.5285 to -0.2110	**<0.0001**
Control 11-15 vs. Davis Rd. [35] 11-15	-0.3942	-0.5530 to -0.2355	**<0.0001**
Control 16-20 vs. Davis Rd. [12] 16-20	-0.2689	-0.4277 to -0.1102	**<0.0001**
Control 16-20 vs. Davis Rd. [20] 16-20	-0.5258	-0.6846 to -0.3671	**<0.0001**
Control 16-20 vs. Davis Rd. [35] 16-20	-0.4777	-0.6365 to -0.3190	**<0.0001**
Control 21-25 vs. Davis Rd. [12] 21-25	-0.2035	-0.3623 to -0.04475	**0.0017**
Control 21-25 vs. Davis Rd. [20] 21-25	-0.457	-0.6157 to -0.2982	**<0.0001**
Control 21-25 vs. Davis Rd. [35] 21-25	-0.4085	-0.5673 to -0.2498	**<0.0001**
Control 26-30 vs. Davis Rd. [12] 26-30	-0.1649	-0.3236 to -0.006097	**0.0332**
Control 26-30 vs. Davis Rd. [20] 26-30	-0.4817	-0.6405 to -0.3229	**<0.0001**
Control 26-30 vs. Davis Rd. [35] 26-30	-0.4082	-0.5669 to -0.2494	**<0.0001**

ANOVA (Photomotor Response)

**Alisal Creek**					
ANOVA table	SS	DF	MS	P value	F (DFn, DFd)
Treatment (between columns)	4.053	3	1.351		
Residual (within columns)	108.1	95	1.138	P=0.3189	F (3, 95) = 1.187
Total	112.2	98	n/a		
**Salinas River**					
ANOVA table	SS	DF	MS	P value	F (DFn, DFd)
Treatment (between columns)	0.7733	3	0.2578		
Residual (within columns)	230.1	154	1.494	P=0.9148	F (3, 154) = 0.1725
Total	230.9	157	n/a		

Table S7. November 2019 (after first flush): Water quality parameters measured using a YSI EXO1 multi-parameter water quality sonde. Parameters recorded include ambient water pH, specific conductance, dissolved oxygen, temperature, alkalinity, hardness, and salinity. Boxes with n/a refer to treatments which were no longer measured due to 100% mortality. Test termination data was not taken for Chlorantraniliprole and Imidacloprid single and binary mixture exposures.

**November 2019 Test Initiation**

SiteID	pH (units)	Cond (ms/cm)	DO (mg/L)	Temp (°C)	Alkalinity (CaCO3) mg/L	Hardness (CaCO3) mg/L	Salinity (ppt)
Quail Creek	8.29	0.485	8.43	24.3	110	190	0.2
Alisal Creek	8.2	0.282	7.68	24.1	96	91	0.1
Salinas River	8.24	0.192	8.8	21.2	36	64	0.1

November 2019 48h Water Change

SiteID	pH (units)	Cond (ms/cm)	DO (mg/L)	Temp (°C)	Alkalinity (CaCO3) mg/L	Hardness (CaCO3) mg/L	Salinity (ppt)
Quail Creek	8.24	0.559	8.81	21.2	n/a	n/a	0.3
Alisal Creek	7.62	0.338	7.4	21.3	38	112	0.2
Salinas River	8.11	0.234	8.57	21	38	70	0.1

November 2019 Test Termination

SiteID	pH (units)	Cond (ms/cm)	DO (mg/L)	Temp (°C)	Alkalinity (CaCO3) mg/L	Hardness (CaCO3) mg/L	Salinity (ppt)
Quail Creek	8.29	0.531	8.59	22.1	109	208	0.3
Alisal Creek	8.24	0.486	8.68	22	124	160	0.3
Salinas River	7.68	0.175	8.24	21.9	42	72	0.1

Table S8. November 2019 (after first flush): Results of Repeated Measures ANOVA and Tukey's Multiple comparisons tests for Total Distance Moved, and Photomotor Response. These data sets were then log transformed and analyzed in GraphPad Prism (v8.0) using a one-way ANOVA with a Tukey's Post Hoc test of multiple comparisons.

November 2019 (after first flush)

Repeated Measures ANOVA

Fixed effects (type III)	P value	F (DFn, DFd)
Time Bin	**<0.0001**	F (3, 192) = 13.46
Treatment	**<0.0001**	F (5.299, 647.5) = 16.08
Time Bin x Treatment	>0.9999	F (30, 1222) = 0.2978

Tukey's Multiple Comparison's (Total Distance Moved)

**Tukey's multiple comparisons test**	**Mean Diff.**	**95.00% CI of diff.**	**Adjusted P Value**
Control 6-10 vs. Quail Creek [6] 6-10	0.5267	0.3827 to 0.6707	**<0.0001**
Control 6-10 vs. Quail Creek [12] 6-10	0.07791	-0.06609 to 0.2219	0.8972
Control 6-10 vs. Quail Creek [20] 6-10	0.02417	-0.1198 to 0.1682	>0.9999

Control 11-15 vs. Quail Creek [6] 11-15	0.1615	0.01752 to 0.3055	**0.0129**
Control 11-15 vs. Quail Creek [12]11-15	-0.2066	-0.3506 to -0.06265	**0.0002**
Control 11-15 vs. Quail Creek [20] 11-15	-0.07355	-0.2176 to 0.07044	0.9356
Control 16-20 vs. Quail Creek [6] 16-20	0.1657	0.02174 to 0.3097	**0.0091**
Control 16-20 vs. Quail Creek [12] 16-20	-0.1418	-0.2858 to 0.002239	0.0587
Control 16-20 vs. Quail Creek [20] 16-20	0.03491	-0.1091 to 0.1789	>0.9999
Control 21-25 vs. Quail Creek [6] 21-25	0.183	0.03898 to 0.3270	**0.002**
Control 21-25 vs. Quail Creek [12] 21-25	-0.06845	-0.2124 to 0.07555	0.9663
Control 21-25 vs. Quail Creek [20] 21-25	0.05128	-0.09272 to 0.1953	0.9987
Control 26-30 vs. Quail Creek [6] 26-30	0.09062	-0.05338 to 0.2346	0.7204
Control 26-30 vs. Quail Creek [12] 26-30	0.01855	-0.1346 to 0.1717	>0.9999
Control 26-30 vs. Quail Creek [20] 26-30	0.0347	-0.1093 to 0.1787	>0.9999
Control 6-10 vs. Hartnell Rd. [6] 6-10	0.3162	0.1480 to 0.4844	**<0.0001**
Control 6-10 vs. Hartnell Rd. [12] 6-10	0.2965	0.1283 to 0.4646	**<0.0001**
Control 6-10 vs. Hartnell Rd. [20] 6-10	0.2355	0.06730 to 0.4036	**0.0003**
Control 11-15 vs. Hartnell Rd. [6] 11-15	-0.007048	-0.1752 to 0.1611	>0.9999
Control 11-15 vs. Hartnell Rd. [12] 11-15	-0.08719	-0.2554 to 0.08098	0.9274
Control 11-15 vs. Hartnell Rd. [20] 11-15	0.1751	0.006961 to 0.3433	**0.0322**
Control 16-20 vs. Hartnell Rd. [6] 16-20	0.006858	-0.1613 to 0.1750	>0.9999
Control 16-20 vs. Hartnell Rd. [12] 16-20	-0.1223	-0.2905 to 0.04583	0.464
Control 16-20 vs. Hartnell Rd. [20] 16-20	0.2782	0.1100 to 0.4463	**<0.0001**
Control 21-25 vs. Hartnell Rd. [6] 21-25	0.08589	-0.08228 to 0.2541	0.936
Control 21-25 vs. Hartnell Rd. [12] 21-25	0.01288	-0.1553 to 0.1811	>0.9999
Control 21-25 vs. Hartnell Rd. [20] 21-25	0.171	0.002847 to 0.3392	**0.0419**
Control 26-30 vs. Hartnell Rd. [6] 26-30	0.1503	-0.01782 to 0.3185	0.1385
Control 26-30 vs. Hartnell Rd. [12] 26-30	-0.02453	-0.1927 to 0.1436	>0.9999
Control 26-30 vs. Hartnell Rd. [20] 26-30	0.1708	0.002645 to 0.3390	**0.0424**
Control 6-10 vs. Davis Rd. [6] 6-10	0.331	0.1450 to 0.5171	**<0.0001**
Control 6-10 vs. Davis Rd. [12] 6-10	0.2309	0.04487 to 0.4169	**0.0023**
Control 6-10 vs. Davis Rd. [20] 6-10	0.3882	0.2022 to 0.5743	**<0.0001**
Control 6-10 vs. Davis Rd. [100] 6-10	0.8842	0.6982 to 1.070	**<0.0001**
Control 11-15 vs. Davis Rd. [6] 11-15	0.05101	-0.1350 to 0.2370	>0.9999
Control 11-15 vs. Davis Rd. [12] 11-15	-0.0009392	-0.1870 to 0.1851	>0.9999
Control 11-15 vs. Davis Rd. [20] 11-15	0.1641	-0.02193 to 0.3501	0.1648
Control 11-15 vs. Davis Rd. [100] 11-15	0.4229	0.2369 to 0.6089	**<0.0001**
Control 16-20 vs. Davis Rd. [6] 16-20	0.0968	-0.08924 to 0.2828	0.9559
Control 16-20 vs. Davis Rd. [12] 16-20	0.02709	-0.1589 to 0.2131	>0.9999
Control 16-20 vs. Davis Rd. [20] 16-20	0.1152	-0.07081 to 0.3013	0.8017
Control 16-20 vs. Davis Rd. [100] 16-20	0.5881	0.4020 to 0.7741	**<0.0001**
Control 21-25 vs. Davis Rd. [6] 21-25	0.1435	-0.04256 to 0.3295	0.3933
Control 21-25 vs. Davis Rd. [12] 21-25	0.1027	-0.08338 to 0.2887	0.9221
Control 21-25 vs. Davis Rd. [20] 21-25	0.166	-0.02003 to 0.3520	0.1502
Control 21-25 vs. Davis Rd. [100] 21-25	0.6969	0.5109 to 0.8830	**<0.0001**

Control 26-30 vs. Davis Rd. [6] 26-30	0.203	0.01700 to 0.3891	**0.017**
Control 26-30 vs. Davis Rd. [12] 26-30	0.09661	-0.08942 to 0.2827	0.9568
Control 26-30 vs. Davis Rd. [20] 26-30	0.1573	-0.02874 to 0.3433	0.2263
Control 26-30 vs. Davis Rd. [100] 26-30	0.5994	0.4133 to 0.7854	**<0.0001**

ANOVA (Photomotor Response)

**Quail Creek**					
ANOVA table	SS	DF	MS	P value	F (DFn, DFd)
Treatment (between columns)	23.72	3	7.905		
Residual (within columns)	209.8	150	1.398	**P=0.0011**	F (3, 150) = 5.653
Total	233.5	153	n/a		
**Alisal Creek**					
ANOVA table	SS	DF	MS	P value	F (DFn, DFd)
Treatment (between columns)	13.81	3	4.604		
Residual (within columns)	198.1	124	1.598	**P=0.0386**	F (3, 124) = 2.881
Total	212	127	n/a		
**Salinas River**					
ANOVA table	SS	DF	MS	P value	F (DFn, DFd)
Treatment (between columns)	21.15	4	5.288		
Residual (within columns)	238.3	173	1.378	**P=0.0051**	F (4, 173) = 3.839
Total	259.5	177	n/a		

Table S9. CHL/IMI acute exposures: Water quality parameters measured using a YSI EXO1 multi-parameter water quality sonde. Parameters recorded include ambient water pH, specific conductance, dissolved oxygen, temperature, alkalinity, hardness, and salinity. Boxes with n/a refer to treatments which were no longer measured due to 100% mortality. Test termination data was not taken for Chlorantraniliprole and Imidacloprid single and binary mixture exposures.

**Chlorantraniliprole and Imidacloprid Test Initiation**							
Treatment ID	pH (units)	Cond (ms/cm)	DO (mg/L)	Temp (°C)	Alkalinity (CaCO3) mg/L	Hardness (CaCO3) mg/L	Salinity (ppt)
Control	8.25	0.574	8.84	20	124	194	0.3
Solvent Control	8.33	0.552	8.84	20	118	182	0.3
CHL Low	8.26	0.55	8.82	20	124	184	0.3
CHL High	8.39	0.584	8.86	20	125	192	0.3
IMI Low	8.41	0.586	8.67	20	125	186	0.3
IMI High	8.4	0.563	8.88	20	115	184	0.3
CHL High IMI High	8.37	0.605	8.77	20	118	190	0.3

CHL High IMI Low	8.39	0.592	8.74	20	117	196	0.3
CHL Low IMI High	8.34	0.576	8.78	20	120	184	0.3
CHL Low IMI Low	8.32	0.579	8.77	20	123	184	0.3
**Chlorantraniliprole and Imidacloprid 48h Water Change**							
Treatment ID	pH (units)	Cond (ms/cm)	DO (mg/L)	Temp (°C)	Alkalinity (CaCO3) mg/L	Hardness (CaCO3) mg/L	Salinity (ppt)
Control	7.76	559	8.8	21.9	102	170	0.3
Solvent Control	8.36	510	9.1	21.1	100	170	0.3
CHL Low	8.42	492	9.7	21.7	106	162	0.3
CHL High	8.42	505	9.09	21	110	168	0.3
IMI Low	8.27	517	8.97	21	106	164	0.3
IMI High	8.34	499	8.81	21.2	108	168	0.3
CHL High IMI High	8.32	0.524	8.91	21	106	176	0.3
CHL High IMI Low	8.45	0.505	8.96	20.6	110	164	0.3
CHL Low IMI High	8.3	0.54	8.94	20.7	106	182	0.3
CHL Low IMI Low	8.25	0.524	9.05	21	106	168	0.3

Table S10. CHL/IMI acute exposures: Mean survival of *D. magna* after 48h and 96h of exposure to CHL, IMI, and binary mixtures of CHL/IMI. Treatments with significant mortality are shown in bolded text. P-values are as reported along with nonsignificant mortality (ns = p > 0.05).

Treatment (µg/L)	48h Survival		96h Survival	
**CHL**				
DIEPAMHR	97.50%	ns	97.50%	ns
Solvent Control	100.00%	ns	100.00%	ns
0.025 CHL	100.00%	ns	100.00%	ns
0.05 CHL	100.00%	ns	100.00%	ns
0.10 CHL	95.00%	ns	90.00%	ns
0.500 CHL	100.00%	ns	90.00%	ns
1.000 CHL	100.00%	ns	100.00%	ns
10.000 CHL	0.00%	**< 0.0001**	0.00%	**< 0.0001**
**IMI**				
DIEPAMHR	91.30%	ns	83.10%	ns
0.025 IMI	97.50%	ns	97.50%	ns

0.05 IMI	100.00%	ns	100.00%	ns
0.10 IMI	100.00%	ns	100.00%	ns
0.500 IMI	100.00%	ns	100.00%	ns
1.000 IMI	100.00%	ns	100.00%	ns
10.000 IMI	100.00%	ns	100.00%	ns
**CHL/IMI**				
DIEPAMHR	100.00%	ns	100.00%	ns
Solvent Control	100.00%	ns	100.00%	ns
0.025 IMI+CHL	100.00%	ns	100.00%	ns
0.500 IMI+CHL	100.00%	ns	80.40%	**0.0001**
10.000 IMI+CHL	0.00%	**< 0.0001**	0.00%	**< 0.0001**

Table S11. CHL/IMI acute exposures: Results of Repeated Measures ANOVA and Tukey's Multiple comparisons tests for Total Distance Moved, and Photomotor Response. These data sets were then log transformed and analyzed in GraphPad Prism (v8.0) using a one-way ANOVA with a Tukey's Post Hoc test of multiple comparisons.

**CHL/IMI**			
Repeated Measures ANOVA			
Fixed effects (type III)	P value	F (DFn, DFd)	
Time Bin	**<0.0001**	F (3, 888) = 20.29	
Treatment	**<0.0001**	F (7.098, 700.3) = 5.514	
Time Bin x Treatment	0.9952	F (27, 888) = 0.4321	
Tukey's Multiple Comparison's (Total Distance Moved)			
**Tukey's multiple comparisons test**	**Mean Diff.**	**95.00% CI of diff.**	**Adjusted P Value**
Control 6-10 vs. IMI High 6-10	0.3235	0.1683 to 0.4787	**<0.0001**
Control 6-10 vs. IMI Low 6-10	0.5715	0.4163 to 0.7266	**<0.0001**
Control 11-15 vs. IMI High 11-15	0.2151	0.05989 to 0.3702	**0.0007**
Control 11-15 vs. IMI low 11-15	0.2891	0.1339 to 0.4443	**<0.0001**
Control 16-20 vs. IMI High 16-20	0.3644	0.2092 to 0.5196	**<0.0001**
Control 16-20 vs. IMI low 16-20	0.3158	0.1606 to 0.4710	**<0.0001**
Control 21-25 vs. IMI High 21-25	0.2941	0.1389 to 0.4493	**<0.0001**
Control 21-25 vs. IMI low 21-25	0.3338	0.1786 to 0.4890	**<0.0001**
Control 26-30 vs. IMI High 26-30	0.2921	0.1369 to 0.4473	**<0.0001**
Control 26-30 vs. IMI low 26-30	0.3511	0.1959 to 0.5063	**<0.0001**
Solvent Control 6-10 vs. CHL High 6-10	0.1426	-0.01376 to 0.2991	0.1097
Solvent Control 6-10 vs. CHL Low 6-10	0.173	0.01657 to 0.3294	**0.0174**
Solvent Control 11-15 vs. CHL High 11-15	-0.1249	-0.2813 to 0.03153	0.2606
Solvent Control 11-15 vs. CHL Low 11-15	-0.07546	-0.2319 to 0.08095	0.9207
Solvent Control 16-20 vs. CHL High 16-20	0.03791	-0.1185 to 0.1943	>0.9999
Solvent Control 16-20 vs. CHL Low 16-20	0.03464	-0.1218 to 0.1910	>0.9999

Solvent Control 21-25 vs. CHL High 21-25	0.05305	-0.1034 to 0.2095	0.9962
Solvent Control 21-25 vs. CHL Low 21-25	0.1188	-0.03756 to 0.2753	0.3345
Solvent Control 26-30 vs. CHL High 26-30	-0.01815	-0.1746 to 0.1383	>0.9999
Solvent Control 26-30 vs. CHL Low 26-30	0.1079	-0.04847 to 0.2643	0.4923
Solvent Control 21-25 vs. CHL High*IMI High 21-25	0.01044	-0.1799 to 0.2008	>0.9999
Solvent Control 21-25 vs. CHL High * IMI Low 21-25	-0.1147	-0.3051 to 0.07566	0.8378
Solvent Control 21-25 vs. CHL Low * IMI High 21-25	0.2035	0.01317 to 0.3939	**0.0223**
Solvent Control 21-25 vs. CHL Low * IMI Low 21-25	0.06217	-0.1282 to 0.2525	>0.9999
Solvent Control 26-30 vs. CHL High*IMI High 26-30	0.01938	-0.1710 to 0.2097	>0.9999
Solvent Control 26-30 vs. CHL High * IMI Low 26-30	-0.1583	-0.3486 to 0.03208	0.2536
Solvent Control 26-30 vs. CHL Low * IMI High 26-30	0.1053	-0.08503 to 0.2957	0.9202
Solvent Control 26-30 vs. CHL Low * IMI Low 26-30	0.1048	-0.08555 to 0.2952	0.9237

ANOVA (Photomotor Response)

**IMI**					
ANOVA table	SS	DF	MS	P value	F (DFn, DFd)
Treatment (between columns)	14.55	2	7.273		
Residual (within columns)	63.97	68	0.9408	**P=0.0009**	F (2, 68) = 7.731
Total	78.52	70	n/a		
**CHL**					
ANOVA table	SS	DF	MS	P value	F (DFn, DFd)
Treatment (between columns)	5.515	2	2.758		
Residual (within columns)	93.83	68	1.38	P=0.1434	F (2, 68) = 1.998
Total	99.35	70			
**CHL/IMI Binary Mixtures**					
ANOVA table	SS	DF	MS	P value	F (DFn, DFd)
Treatment (between columns)	3.038	4	0.7594		
Residual (within columns)	114	108	1.055	P=0.5803	F (4, 108) = 0.7197
Total	117	112	n/a		
